# A Geospatial database of gastric cancer patients and associated potential risk factors including lifestyle and air pollution

**DOI:** 10.1186/s13104-021-05506-x

**Published:** 2021-03-09

**Authors:** Fatemeh Hashemi Amin, Mahtab Ghaemi, Sayyed Mostafa Mostafavi, Ladan Goshayeshi, Khadijeh Rezaei, Mehdi Vahed, Behzad Kiani

**Affiliations:** 1 Department of Medical Informatics, School of Medicine, Mashhad University of Medical Sciences, Mashhad, Iran; 2 Department of Internal Medicine, School of Medicine, Mashhad University of Medical Sciences, Mashhad, Iran; 3 Department of Gastroenterology and Hepatology, School of Medicine, Mashhad University of Medical Sciences, Mashhad, Iran; 4 Department of Health, Mashhad University of Medical Sciences, Mashhad, Iran; 5 Department of Medical Informatics, School of Medicine, Mashhad University of Medical Sciences, Mashhad, Iran

**Keywords:** Gastric cancer, Geographical information systems, GIS, Geospatial analysis, Dietary habits, Air pollutants, Environmental factors, Stomach cancer

## Abstract

**Objectives:**

Gastric cancer (GC) is a multifactorial disease and the fifth most frequent diagnosed cancer worldwide. It accounts for one third of cancer-related mortalities. Geospatial analysis using geographical information systems (GIS) can provide an efficient solution to identify spatial disparities associated with GC. As such, GIS enables policymakers to control cancer in a better way and identify the regions where interventions are needed. This study aims to publish a comprehensive dataset, which was applied to conduct a spatial analysis of GC patients in the city of Mashhad, Iran.

**Data description:**

We provide a personal geodatabase, a Microsoft Access database that can store, query, and manage both spatial and non-spatial data, which contains four feature classes. “Male_Stomach_Cancer_Patients” and “Female_Stomach_Cancer_Patients” are point feature classes, which show the age and geographical location of 1156 GC cancer patients diagnosed between 2014 and 2017. “Air_Polution_Mashhad” is another point feature class that reveals the amount of six air pollutants, which was taken from Mashhad Environmental Pollutants Monitoring Center between 2017 and 2018. Finally, “Stomach_Cancer_and_Risk_Factors” is a polygon feature class of neighborhood division of Mashhad, consisting of contributor risk factors including dietary habits, smoking, alcohol use, body mass index and population by age groups for all 165 city neighborhoods.

## Objective

Gastric cancer (GC), also known as stomach cancer, is classified as the fifth most frequent diagnosed cancer among both genders and is the third leading cause of cancer mortality [1]. According to GLOBOCAN2018, more than 10.6% of different types of cancer cases in Iran are associated with GC. It also contributed to 16.1% of all cancer-related deaths and accounted for the most common cancer-related mortality [2]. The major GC risk factors include alcohol drinking, physical inactivity, chronic infections, gender, age, medical history, smoking and unhealthy eating habits [3–6]. Furthermore, the association between environmental risk factors and GC is widely reported in the literature [7–12]. The presence of significant geographical disparities across the world is one of this tumor's epidemiological traits. This implication indicates that environmental exposures can play a key role in the uncertain carcinogenesis of GC [13].

We conducted a spatial analysis of GC incidence at the neighborhoods level in the city of Mashhad, Iran. Dietary habits, smoking, alcohol drinking, BMI, and air pollution were considered in the model. In this study, we aim to offer a comprehensive integrated geodatabase. This geodatabase is a practical tool for further investigation in future spatial analysis of GC incidence.

## Data description

Geospatial approaches and, in particular, GIS describe the spread and etiology of different types of disease. Moreover, they can provide useful strategies for disease control. An important topic of research is the association between geography and cancer incidence, where GIS applications play an important role [14–16]. GIS tools have the potential to accurately measure healthcare resources, track possible regional improvements in disease outcomes and also identify potential differences in cancer care [17, 18].

In this study, a personal geodatabase was created to store all the data files (feature classes). We obtained the data from four different databases. The data of cancer registry of Khorasan-Razavi Province between March 2014 and March 2017 was extracted to obtain individual GC cases. This dataset contains 1156 records of GC cases along with their age, gender and geographical locations in Mashhad, Iran. The address of GC patients was geocoded and this data is made available in “Male_Stomach_Cancer_Patients” and “Female_Stomach_Cancer_Patients” feature classes in the geodatabase (Data file 1). Regarding the patients’ privacy, the point data of patients were randomly moved within a 500 m. The Mashhad Municipal Council provided the neighborhood divisions and their population into different age groups. The age group interval was estimated at five-year intervals for males and females. This data is made available in the “Stomach_Cancer_and_Risk_Factors” feature class stored in the geodatabase (data file 1).

Data on potential risk factors including Body Mass Index (BMI), smoking, alcohol drink, total intake of red meat, processed meat, fruit, vegetable, salt and smoked food were derived from the MASHHAD Cohort study dataset, containing 6388 records [19]. We calculated the percentage of alcoholic and smoker individuals for each neighborhood. Fields regarding intake of vegetables and fruit were measured in terms of grams per day and consumption of red meat and processed meat were calculated in grams per week. The total amount of smoked food consumed per month was recorded in the smoked food field for every neighborhood. “Stomach_Cancer_and_Risk_Factors” reports this data.

Data regarding amount of six air pollutants was prepared by Mashhad Environmental Pollutants Monitoring Center, consisting Ozone (${O}_{3}$), Particulate matter (PM10, PM2.5), Nitrogen Dioxide (${NO}_{2}$), Carbon Monoxide (CO), Sulfur Dioxide (${SO}_{2}$) between March 2017-March 2018. Spatial interpolation method was employed by ArcGIS 10.6 so as to estimate the amount of air pollutants for each neighborhood where no station was available to calculate the actual amount of particles. These data are available from the “Air_Polution_Mashhad” feature class stored in the geodatabase (data file 1).

To prepare the geodatabase, the parcel layer of Mashhad was considered as the base layer. Then, the GC patients layer, Persian Cohort layer and heavy metal layer were linked by performing spatial joining. The final layer was a polygon layer with an attribute table containing demographic information, risk factors of Persian Cohort database, characteristics of cancer patients and data related to amount of air pollutants for the neighborhoods of Mashhad. In the Persian Cohort database, the amount of salt consumed by each person was specified by assigning 1 to low salt, 2 to medium salt and 3 to high salt. In the final attribute table, the sum of these numbers in each neighborhood was calculated.

We utilized a Geographical Weighted Regression (GWR) model to explore which risk factors were more related to GC incidence in each neighborhood. GWR can be applied for spatial non-stationary parameter recognition by local parameter estimation [20]. Our dataset can be applied in further research in order to assess the association between other potential risk factors and GC incidence. In addition, the provided dataset is useful for those who attempt to investigate the relation of these available risk factors and other kinds of cancers occurrence. As a result of performing these kinds of analyses, we can mention the impacts of them on implementing more efficient cancer prevention plans and developing some new strategies to reduce the huge burden of cancers. These strategies can be specified for each neighborhood in urban areas. For example, in one neighborhood educating people to improve their dietary habits can be essential but in another neighborhood, reducing air pollution can be the first priority.

## Limitations

The data of life style factors aggregated into neighborhood levels were obtained through the PERSIAN Cohort study [19], which is an institutional cohort study. This means that we used a specific sample of the general population, employees of government centers, to estimate life style factors for each neighborhood. This might not be a representative sample of the total population. However, this is the best data available for determining life style factors at neighborhood level in the city of Mashhad, Iran.

**Table 1:** Overview of data files/data sets

Label	Name of data file/data set	File types (file extension)	Data repository and identifier (DOI or accession number)
Data file 1	Stomach_Cancer_Mashhad	MS Access file (.mdb)	*Harvard Dataverse* (https://doi.org/10.7910/DVN/SOQM4D) [21]

## Data Availability

The data described in this data note can be freely and openly accessed on the *Harvard Dataverse* under (https://doi.org/10.7910/DVN/SOQM4D) [21]. Please see Table 1 for details and link to the data.

## Abbreviations

GC: Gastric cancer
GIS: Geographical information systems
